# Temporal trends of preterm birth in Shenzhen, China: a retrospective study

**DOI:** 10.1186/s12978-018-0477-8

**Published:** 2018-03-13

**Authors:** Changchang Li, Zhijiang Liang, Michael S. Bloom, Qiong Wang, Xiaoting Shen, Huanhuan Zhang, Suhan Wang, Weiqing Chen, Yan Lin, Qingguo Zhao, Cunrui Huang

**Affiliations:** 1 Department of Health Policy and Management, School of Public Health, Sun Yat-sen University, 74 Zhongshan Road #2, Guangzhou, 510080 China; 2 Guangzhou Key Laboratory of Environmental Pollution and Health Risk Assessment, School of Public Health, Sun Yat-sen University, 74 Zhongshan Road #2, Guangzhou, 510080 China; 3 Department of Biostatistics and Epidemiology, School of Public Health, Sun Yat-sen University, 74 Zhongshan Road #2, Guangzhou, 510080 China; 4 Department of Public Health, Guangdong Women and Children Hospital, 521, 523 Xing Nan Street, Guangzhou, 511442 China; 5 Departments of Environmental Health Sciences and Epidemiology and Biostatistics, University at Albany, State University of New York, Rensselaer, USA; 6 Center for Reproductive Medicine, The First Affiliated Hospital of Sun Yat-sen University, 74 Zhongshan Road #2, Guangzhou, 510080 China; 7 Department of Children Health Care, Shenzhen Women and Children Hospital, Shenzhen, China

**Keywords:** Preterm birth, Incidence rate, Temporal trend, Medically induced preterm birth, Spontaneous preterm birth, China

## Abstract

**Background:**

Preterm birth is the leading cause of child mortality under 5 years of age. Temporal trends in preterm birth rates are highly heterogeneous among countries and little information exists for China. To address this data gap, we investigated annual changes in preterm birth incidence rate and explored potential determinants of these changes in Shenzhen, China.

**Methods:**

A total of 1.4 million live births, during 2003-2012, were included from the Shenzhen birth registry. Negative-binominal regression models were used to estimate the annual percent changes in incidence. To identify the potential determinants behind temporal trends, we estimated the contribution of each changing risk factor to changes in rate by calculating the difference in population-attributable risk fraction.

**Results:**

Annual preterm birth incidence rates increased by 0.94% (95% CI 0.30%, 1.58%) overall, 3.60% (95% CI 2.73%, 4.48%) for medically induced, and 3.13% (95% CI 1.01%, 5.31%) for preterm premature rupture of membranes, but decreased by 2.34% (95% CI 1.62%, 3.06%) for spontaneous preterm labor. Higher maternal educational attainment (0.20 rate increase), lower proportion of inadequate prenatal care (0.15 rate reduction), more multipara (0.08 rate reduction), decreased proportion of preeclampsia or eclampsia (0.05 rate reduction), and larger proportion of young and older pregnant women (0.04 rate increase) were significant contributors to the overall change over time. Contributions of changing risk factors were different between preterm birth subtypes.

**Conclusions:**

Preterm birth rate in Shenzhen, China increased overall during 2003-2012, although trends varied across three preterm birth subtypes. The rising rates were associated with changes in maternal education and age.

**Electronic supplementary material:**

The online version of this article (10.1186/s12978-018-0477-8) contains supplementary material, which is available to authorized users.

## Plain English summary

Complications from preterm birth (PTB) is the leading cause of neonatal and child mortality worldwide. Numerous studies have reported changes in PTB incidence over the past two decades. These finding showed that the temporal trends in PTB rates are highly heterogeneous among countries, but there is little information available for China.

China has the second greatest number of PTBs worldwide, with large disparities in PTB rates across different regions of the country. To better understand the temporal trends in PTB rates in mainland China, this study investigated changes in PTB rates by clinical subtype and explore potential determinants of the changes in Shenzhen, China.

Based on the data analysis, we found that preterm birth rate increased in Shenzhen between 2003 and 2012, yet with varied trends among three PTB clinical subtypes. In detail, incidence rates increased in late preterm and medically induced preterm birth, but decreased in preterm birth due to spontaneous preterm labor. Maternal education, parity and prenatal care visits played important roles in determining secular trends for PTB rates. In summary, the Shenzhen findings provided complementary evidence to confirm the increasing trends of PTB rates in mainland China. Moreover, this study also suggested that advanced and highly educated pregnant women should be the key target population groups for future clinical intervention and public health prevention strategies in developed area of China.

## Background

Complications from preterm birth (PTB) is the leading cause of neonatal and child mortality worldwide [1]. Globally, it was estimated that 15 million babies yearly, were delivered preterm, which caused one million deaths in children under 5 years of age in 2013 [1, 2]. In addition to increased mortality, PTB infants are at higher risk for suffering chronic health conditions, and neurodevelopmental and learning impairment [3]. Preterm birth introduces enormous physical, psychological and economic costs. A study from Canada indicated that total national cost corresponding to PTB was at least $587.1 million in 2014. The cost per infant over the first 10 years of life was estimated to be $67,467 for early preterm births, $52,796 for moderate preterm births, and $10,010 for late preterm births [4]. Therefore, even a modest reduction in PTB would make for substantially reduced short and long-term costs.

The investigation of temporal trends in PTB rates is essential to inform policy and to design interventions for reducing the burden of PTB. Numerous prior studies have reported changes in PTB incidence over the past two decades. These findings showed that the temporal trends in PTB rates are highly heterogeneous among countries [2, 5]. A global study of 65 developed, Latin America, and Caribbean countries reported higher PTB rates for 2010 than for 1990, although PTB rates were stable for 14 countries, and 3 countries (Croatia, Ecuador, and Estonia) had a decline [2]. Findings from European countries suggested the PTB rate in Austria increased from 1996 to 2004, but then declined slightly between 2004 and 2008 [5]. Notably, variations in PTB clinical subtypes (spontaneous PTB and medically induced PTB) were also highly heterogeneous among different countries [5]. Factors possibly associated with changes in PTB rates include changes in obstetric population characteristics (e.g. older) and risk factors (e.g. multiple gestations), implementation of specific clinical practices (e.g. use of vaginal progesterone), and changes in public health policies and regulations (e.g. smoking bans in public places) [6, 7].

Existing studies of PTB rates have mainly focused on populations in Europe and North America, yet there is little information available for China [2, 5]. After India, China has the highest number of PTBs worldwide, with large disparities in PTB rates across different regions of the country [2, 8]. The incidence of preterm birth was higher in low-income regions than in high-income regions. The highest incidences were recorded in Southwest China and Northeast China. Only two previous studies investigated temporal trends in Chinese PTB rates during the past decades [9, 10], and the conclusions were inconsistent. PTB rates overall increased in mainland China’s Hubei Province, but remained constant in Hong Kong. In an extended analysis, Hui and colleagues [10] suggested that the stable PTB rates in Hong Kong resulted from a pattern of decreasing preterm birth due to spontaneous preterm labor (S-PTB) coupled to increasing preterm birth following premature rupture of membranes (PROM-PTB). However, the trends in mainland China PTB subtypes remain unclear.

Identifying risk factors specific to PTB subtypes will assist clinicians and policymakers in designing interventions to prevent PTB. Hence, a good knowledge of temporal trends in the rates of PTB subtypes and the reasons behind changing rates may enhance PTB prevention [6, 11, 12]. To better understand the temporal trends in PTB rates in mainland China, this study aimed to investigate changes in PTB rates by clinical subtype and to explore potential determinants of the changes in Shenzhen, China.

## Methods

### Study design and setting

We conducted a retrospective cohort study of births in Shenzhen, which located in Guangdong Province in southern China. Shenzhen is the first Special Economic Zone in China, stemming from China’s economic reform in 1980s. It is a megacity with a total population of about 11.9 million. During the past 30 years, the population of Shenzhen has experienced significant socioeconomic and health changes, reflecting the typical development of mainland China. Thus, Shenzhen provides an excellent opportunity to explore the drivers behind health changes in mainland China.

### Data collection

We used the Shenzhen Birth Registry Database to capture data for all live births from January 1, 2003 to December 31, 2012 (*n* = 1.42 million).This birth registry database covers all midwifery clinics and hospitals, allowing for accurate PTB rate calculation. Furthermore, this system connects to a city-wide maternal and children health information system, what also allowed for capture of medical record data, including demographic and clinical information for both mother and newborn. The high validity and reliability of data from the Shenzhen Birth Registry Database was previously described [13].

To minimize variability in reporting over the study period, we excluded births: (1) Missing gestational (0.02%) or maternal (0.01%) ages; (2) With maternal age < 13 years or > 50 years (1.87%); or, (3) With gestational age < 22 weeks or > 46 weeks according to the distribution of gestational ages (0.36%) [14]. The flow of study population selection was shown in Additional file 1: Figure S1.

### Measures

We collected all variables available from the electronic medical record, and selected the variables for inclusion as PTB risk factors based on the literature [3]. We extracted pregnancy and birth data for each live birth including date of birth, date of mother’s last menstrual period (LMP), infant sex (male, female, hermaphrodite), delivery mode (vaginal, cesarean section), parity (0, ≥1), gestational hypertension (yes/ no), preeclampsia or eclampsia (yes/no), and number of prenatal care visits. The number of prenatal care visits was transformed into the adequacy of prenatal care utilization (APNCU) index [15], by calculating the ratio between the actual number of visits and the recommended number. According to the recommendation by the Institute for Clinical Systems Improvement (ICSI), a pregnant woman should be examined four times for the first 28 weeks of pregnancy, five times for 32 weeks, six times for 36 weeks, and 7-11 times for 37-41 weeks of pregnancy [16]. We classified the index into four groups: inadequate (< 50%), intermediate (50-79%), appropriate (80-109%) and appropriate plus (≥ 110%).

We also extracted maternal sociodemographic characteristics and chronic maternal conditions data. We categorized maternal education as no high school, high school and college, bachelor, and postgraduate degree. Chronic maternal conditions included clinically diagnosed hypertension, hepatopathy, nephropathy, and heart disease.

### Classification of preterm birth subtypes

We defined PTB as live born infants at less than 37 completed weeks of gestation from the date of LMP, or corrected by first trimester ultrasound if discrepant by more than 7 days. We classified PTB into spontaneous preterm birth and medically induced preterm birth (MI-PTB) according to clinical presentation, and then categorized spontaneous preterm births as preterm premature rupture of membranes (PROM-PTB) and preterm labor (S-PTB). The classification criteria were as follows: (1) MI-PTB, defined as labor induction and/or elective cesarean section without PROM; (2) PROM-PTB, regardless of delivery mode or induction and status; and, (3) S-PTB, which included all non-PROM associated vaginal deliveries. Based on this classification scheme, remaining births that did not meet the criteria for PROM-PTB and MI-PTB were categorized as S-PTB [17].

### Statistical analysis

We expressed incidence rate as the number of PTB infants per 100 live births [18]. We calculated annual PTB rates for the entire Shenzhen population and for specific groups defined by PTB subtypes, maternal age, and maternal education. We used negative-binomial regression models to estimate rate ratios (RR), with annual PTB rates operationalized as a count data. RRs were then transformed into the annual percent change (RR-1). We also analyzed changes in proportions of PTB subtypes across time. Risk factors associated with each PTB subtype were identified by using binominal logistic regression models. We included maternal age, and education, infant sex, pregnancy characteristics and chronic maternal conditions in the models. Adjusted odds ratios (AORs) and 95% confidence intervals (95%CI) were calculated to present the risk.

Finally, to analyze the contribution of changing risk factors to changes in PTB rate, we calculated the difference in population-attributable risk fraction (*AF*_*p*_) for each changing risk factor. The Born Too Soon Preterm Prevention Analysis Group used this method to analyze drivers for increasing PTB rates in the U.S. [7]. The process for this approach follows:

i. Identify distributions of each risk factor for 2003-2007 and 2008-2012. We selected the year of 2007 as the cut-off year because Shenzhen PTB rates increased after 2007.
ii. Identify the PTB for every category of each risk factor during 2008-2012, using ORs generated from logistic regression models.
iii. Calculate *AF*_*p*_ for each PTB risk factor, and compute specific *AF*_*p*_ values for 2003-2007 and 2008-2012. We defined *AF*_*p*i_ and *AF*_*p*,_ where, *AF*_*p*i_ is the population attributed risk fraction for exposure category *j* of the (*j* = 1….n) *i*th risk factor, *PF*_*j*_ is the proportion of the total population in exposure category *j* for the *ith* risk factor, *RR*_*j*_ is the risk ratio for the exposure category *j* of the *ith* risk factor, approximated using ORs, and *AF*_*p*_ is the population attributable risk across all risk factors *i* [19]. The formulas were as follows:

1 $$ {AF}_{Pi}=\frac{\sum \limits_{j=1}^n{PF}_j\left({RR}_j-1\right)}{1+\sum \limits_{j=1}^n{PF}_j\left({RR}_j-1\right)} $$

2 $$ {AF}_p=1-\prod \limits_{\mathrm{i}=1}^{\mathrm{n}}\left(1-{AF}_{P\mathrm{i}}\right) $$

Notably, the *AF*_*Pi*_ value of each risk factor for 2003-2007 was calculated by *PF*_*j*_ in 2003-2007 and *RR*_*j*_ in 2008-2012, whereas *AF*_*Pi*_ value for 2008-2012 was a result of *PF*_*j*_ and *RR*_*j*_ in 2008-2012.

iv. Multiply the *AF*_*P*_ by PTB rates in 2008-2012 and subtract the result for 2003-2007 from 2008 to 2012 (Formula 3). The difference was the projected increased in PTB rates between two study periods for each changing risk factor.

3 $$ \mathrm{Projected}\ \mathrm{increase}=A{F}_{P2008-2012}\ast rat{e}_{2008-2012}-A{F}_{P2003-2007}\ast rat{e}_{2008-2012}\kern0.5em $$

### Sensitivity analysis

We performed a sensitivity analysis to ensure the robustness of our findings. Recognizing uncertainty in linear trends, we included year of delivery as a dummy variable into a negative-binominal regression model to examine changes in PTB rates by individual year.

All the analyses were conducted using R software (version 3.2.4; R Foundation for Statistical Computing, Vienna, Austria). An alpha level of 0.05 indicated statistical significance for a two-tailed test.

## Results

### Preterm birth rates in Shenzhen during the 10-year period 2003–2012

A total of 1.42 million births were recorded in the Shenzhen Birth Registry Database between 2003 and 2012. After excluding the 32,172 (2.25%) ineligible records and 2135 (0.15%) still births, we included 1.39 million (97.6%) live births in this study. There were 78,252 (5.7%) PTBs with PROM-PTB, S-PTB and MI-PTB accounting for 9.5%, 51.4% and 39.4% of overall PTBs, respectively. PTB rates among different maternal and infant groups are presented in Table 1. Subtype-specific PTB rates appeared to differ by maternal age and education. For example, S-PTB rates were higher in younger mothers and in less educated mothers, but MI-PTB rates were higher in older mothers and more highly educated mothers.

**Table 1:** Descriptive Statistics of All Live Births and Preterm Births (PTB) in Shenzhen, China during 2003–2012

	Live births	Term births		Spontaneous preterm births ^b^				Medically induced preterm birth	
				PROM-PTB		S-PTB			
		N	%	N	%	N	%	N	%
All live birth	1,385,882	1,307,570	94.35	7436	0.54	40,104	2.89	30,712	2.21
Maternal age (years)									
≤ 20	81,436	75,759	93.03	315	0.39	4375	5.37	987	1.21
21-35	1,230,521	1,164,107	94.60	6477	0.53	33,773	2.74	26,164	2.13
≥ 36	73,865	67,704	91.66	644	0.87	1956	2.65	3561	4.82
Maternal education									
Less than high school	599,640	565,404	94.29	2397	0.40	21,037	3.51	10,802	1.80
High school and college	498,618	469,616	94.18	2993	0.60	13,912	2.79	12,097	2.43
Bachelor	263,880	250,133	94.79	1884	0.71	4765	1.81	7098	2.69
Postgraduate	23,684	22,417	94.65	162	0.68	390	1.65	715	3.02
Parity									
0	857,543	808,052	94.23	5277	0.62	25,120	2.93	19,094	2.23
≥ 1	523,788	495,377	94.58	2139	0.41	14,778	2.82	11,494	2.19
Missing data	4491	–	–	–	–	–	–		
APNCU index ^a^									
Inadequate	635,795	590,381	92.86	3711	0.58	27,182	4.28	14,521	2.28
Intermediate	388,153	366,230	94.35	2427	0.63	9716	2.50	9780	2.52
Appropriate	164,493	157,253	95.60	876	0.53	2458	1.49	3906	2.37
Appropriate plus	197,234	193,572	98.14	421	0.21	744	0.38	2497	1.27
Missing	147	–	–	–	–	–	–		
Maternal chronic conditions									
Yes	3152	2629	83.41	80	2.54	102	3.24	341	10.82
No	1,382,670	1,304,941	94.38	7356	0.53	40,002	2.89	30,371	2.20
Gestational hypertension									
Yes	5008	4496	89.78	72	1.44	101	2.02	339	6.77
No	1,380,814	1,303,074	94.37	7364	0.53	40,003	2.90	30,373	2.20
Preeclampsia or eclampsia									
Yes	16,208	12,644	78.01	102	0.63	309	1.91	3153	19.45
No	1,369,614	1,294,926	94.55	7334	0.54	39,795	2.91	27,559	2.01
Infant sex									
Male	752,163	707,283	94.03	4306	0.57	23,432	3.11	17,142	2.28
Female	633,466	600,123	94.74	3130	0.49	16,648	2.63	13,565	2.14
Hermaphrodite	193	164	84.97	0.00	0.00	24	12.44	5	2.59

^a^*APNCU* the adequacy of prenatal care utilization

^b^*PROM-PTB* preterm birth following premature rupture of membranesm, *S-PTB* preterm birth due to spontaneous preterm labor

### Time trends in preterm birth rate

**Fig. 1:**
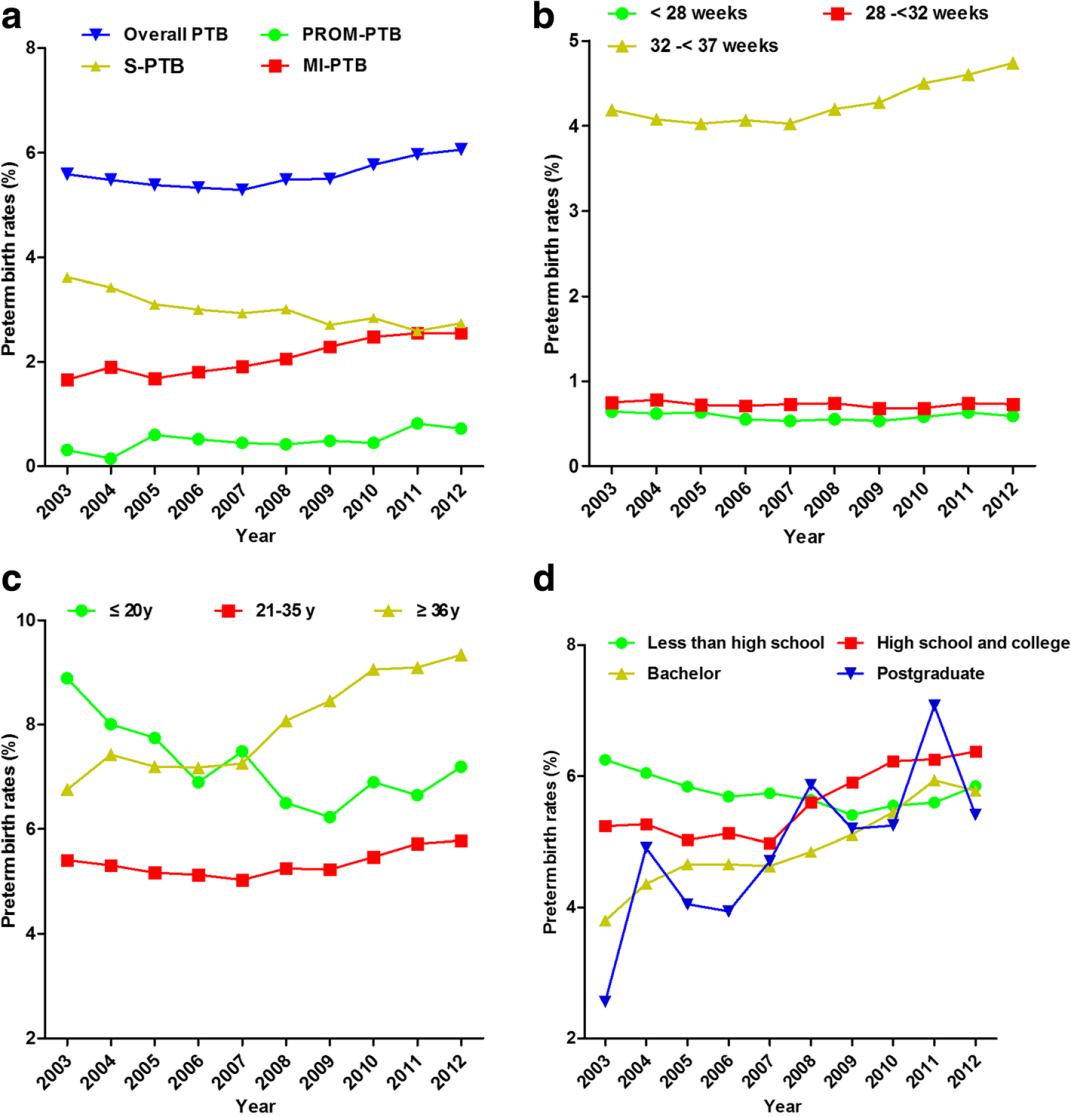
**a** Temporal trends in rates of overall and subtype-specific preterm births (PTB) in Shenzhen China, 2003-2012. *PROM-PTB*, preterm birth following premature rupture of membranes; *MI-PTB*, medically induced preterm birth; *S-PTB*, preterm birth due to spontaneous preterm labor. **b** Preterm birth rates by gestational age. **c** Overall preterm birth rates by maternal age. **d** Overall preterm birth rates by maternal education

Figure 1 shows that PTB rates increased from 5.6% in 2003 to 6.06% in 2012, corresponding to a 0.94% annual rise (95%CI 0.30%, 1.58%) (Additional file 1: Table S1). There were approximately 3.60% (95%CI 2.73%, 4.48%), and 3.13% (95%CI 1.01%, 5.31%) overall increases in MI-PTB and PROM-PTB, respectively, but S-PTB decreased by about 2.34% (95%CI 3.06%, 1.62%) per year during the study period. Time trends for PTB rates by infant gestational age and maternal age and education are also presented in Fig. 1. Significant increasing trends were detected for moderate and late preterm (32- < 37 gestational weeks), older mothers (≥ 36 years) and mothers with higher educational attainment (vs. less than high school). As shown by the sensitivity analysis in Additional file 1: Table S1, the overall PTB rates were lower in 2005-2007 than in 2003, however the differences were not statistically significant as shown in Additional file 1: Table S2. In contrast, overall PTB rates in 2008, 2009, 2010, and 2012 were significantly higher than for 2003 (Additional file 1: Table S2).

### Risk factors for preterm birth by subtype

As described by Table 2, statistically significant risk factors for PTBs included chronic maternal conditions, inadequate prenatal care and male infant sex. In contrast, multipara consistently decreased the risk. The effects of maternal age and education were less consistent across the three PTB subtypes. S-PTB was more likely to be associated with younger maternal age and lower education. We also detected associations between maternal chronic conditions and gestational complications with higher rates of PROM-PTB and MI-PTB, although with lower rates for S-PTB with gestational hypertension and preeclampsia or eclampsia.

**Table 2:** Multivariable Logistic Regression Analysis of Risk Factors for Preterm Birth (PTB) Subtypes in Shenzhen, China, 2003–2012

	Spontaneous preterm birth ^b^				Medically induced preterm birth	
	PROM-PTB		S-PTB			
	*β*	*AOR*^c^ (95% CI)	*β*	*AOR*^c^ (95% CI)	*β*	*AOR*^c^ (95% CI)
Maternal age (years)						
21-35	–	Reference	–	Reference	–	Reference
≤ 20	−0.400	0.670 **(**0.597, 0.753**)**	0.386	1.471 (1.422, 1.522)	−0.539	0.583 (0.546, 0.623)
≥ 36	0.706	2.026 **(**1.863, 2.203**)**	−0.026	0.974 (0.930, 1.021)	0.799	2.224 (2.141, 2.31)
Maternal education						
Bachelor	–	Reference	–	Reference	–	Reference
Less than high school	−0.791	0.454 (0.424, 0.485)	0.074	1.077 (1.040, 1.114)	−0.627	0.534 (0.516, 0.553)
High school and college	−0.334	0.716 (0.681, 0.771)	0.026	1.027 (0.992, 1.063)	−0.252	0.777 (0.753, 0.802)
Postgraduate	−0.071	0.931 (0.792, 1.096)	−0.153	0.858 (0.773, 0.953)	0.092	1.097 (1.013, 1.187)
Parity						
0	**–**	Reference	–	Reference	–	Reference
≥ 1	−0.528	0.590 (0.559, 0.623)	−0.236	0.790 (0.773, 0.807)	−0.114	0.892 (0.870, 0.916)
APNCU index ^a^						
Appropriate	**–**	Reference	–	Reference	–	Reference
Inadequate	0.485	1.625 (1.504, 1.755)	1.075	2.929 (2.806, 3.057)	0.182	1.199 (1.155, 1.245)
Intermediate	0.399	1.490 (1.377, 1.611)	0.536	1.709 (1.634, 1.788)	0.206	1.228 (1.182, 1.276)
Appropriate plus	−1.068	0.344 (0.306, 0.386)	−1.405	0.245 (0.226, 0.266)	−0.735	0.479 (0.455, 0.504)
Maternal chronic conditions						
No	**–**	Reference	–	Reference	–	Reference
Yes	1.755	5.786 (4.617, 7.250)	0.571	1.770 (1.449, 2.162)	1.440	4.222 (3.730, 4.779)
Gestational hypertension						
No	**–**	Reference	–	Reference	–	Reference
Yes	1.008	2.740 (2.166, 3.467)	−0.319	0.727 (0.596, 0.886)	0.864	2.372 (2.109, 2.669)
Preeclampsia or eclampsia						
No	**–**	Reference	–	Reference	–	Reference
Yes	0.227	1.255 (1.029, 1.530)	−0.341	0.711 (0.634, 0.797)	2.378	10.782 (10.339, 11.244)
Infant sex						
Female	**–**	Reference	–	Reference	–	Reference
Male	0.168	1.183 (1.129, 1.239)	0.168	1.183 (1.159, 1.207)	0.082	1.085 (1.060, 1.111)
Hermaphroditism	−8.381	0.002 (0.000, 0.002)	1.459	4.301 (2.784, 6.644)	0.238	1.269 (0.515, 3.129)

^a^*APNCU* the adequacy of prenatal care utilization

^b^*PROM-PTB* preterm birth following premature rupture of membranes, *S-PTB*, preterm birth due to spontaneous preterm labor

^c^*AOR* adjusted odds ratio, *CI *confidence interval

### Contributions of changing risk factors to changes in preterm birth rates

Table 3 describes the percentage of mothers for every category of each risk factor during 2003-2007 and 2008-2012. A notable increase in risk factor incidence occurred among older (≥ 36 years) mothers, mothers with higher educational attainment, multiparous women, and pregnant women with intermediate prenatal care. The changes in the incidence of chronic conditions, gestational hypertension, preeclampsia or eclampsia, and male infant were comparatively modest.

**Table 3:** Distribution of Risk Factors for Preterm Birth (PTB) in Shenzhen, China, 2003- 2012

	Incidence rates (%)	
	2003-2007	2008-2012
Preterm birth ^a^		
Overall-PTB	5.38	5.79
PROM-PTB	0.43	0.59
S-PTB	3.24	2.84
MI-PTB	1.81	2.43
Maternal age (years)		
21-35	90.71	87.78
≤ 20	4.62	6.54
≥ 36	4.66	5.68
Maternal education		
Less than high school	50.11	39.66
High school and college	32.79	37.66
Bachelor	16.26	20.51
Postgraduate	0.83	2.17
Parity		
0	66.57	59.40
≥ 1	33.43	40.10
Missing	0.00	0.50
APNCU index ^b^		
Inadequate	54.81	40.93
Intermediate	14.84	30.14
Appropriate	15.61	14.4
Appropriate plus	14.73	15.3
Missing	0.00	0.02
Maternal chronic conditions		
No	99.81	99.76
Yes	0.19	0.24
Gestational hypertension		
No	99.79	99.56
Yes	0.21	0.44
Preeclampsia or eclampsia		
No	98.67	98.92
Yes	1.33	1.08
Infant sex		
Female	45.13	46.02
Male	54.85	53.97
Hermaphrodite	0.02	0.01

^a^*Overall-PTB* all preterm births, *PROM-PTB* preterm birth following premature rupture of membranes, *S-PTB* preterm birth due to spontaneous preterm labor, *MI-PTB* medically induced preterm birth

^b^*APNCU* the adequacy of prenatal care utilization

**Fig. 2:**
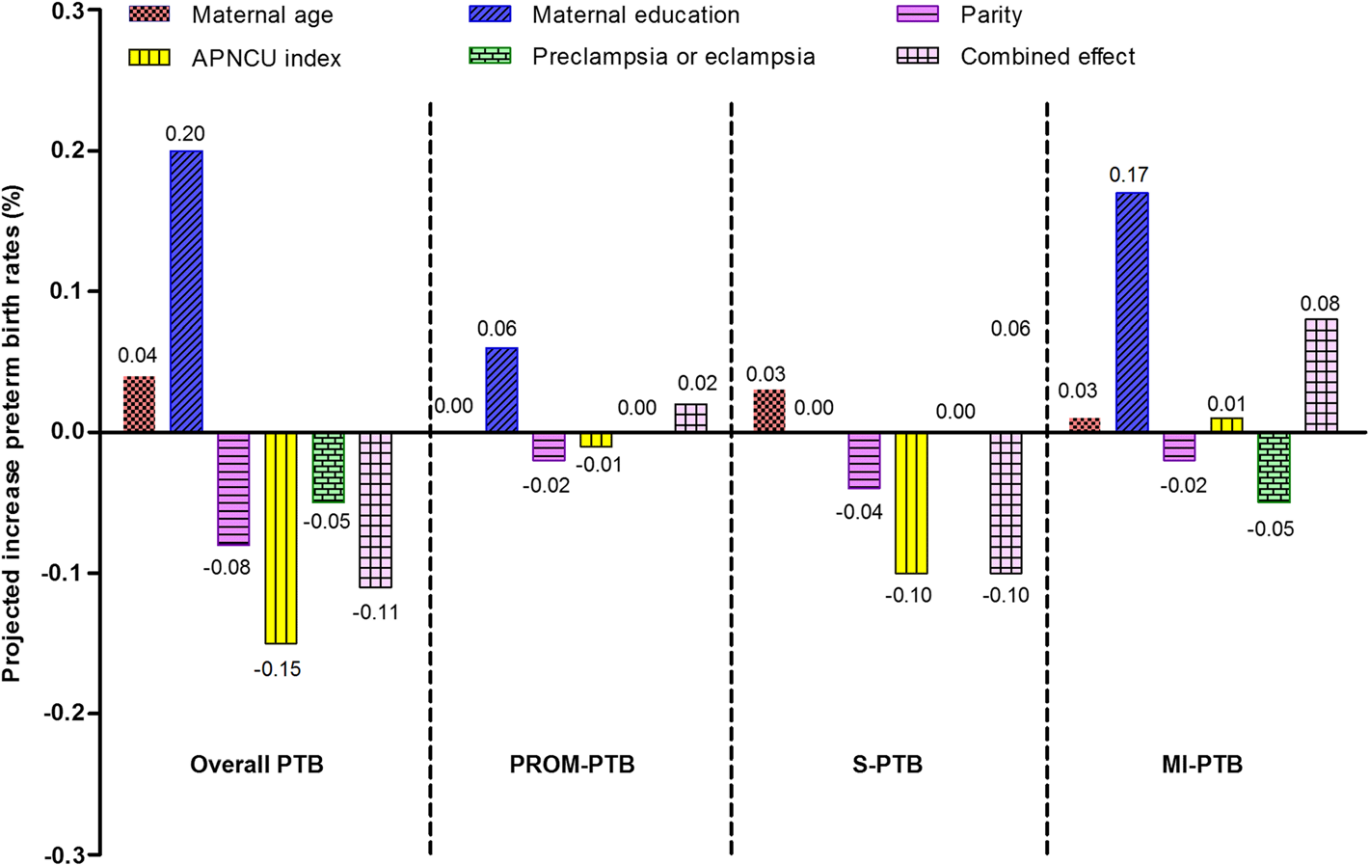
Contributions of changing risk factors to changes in preterm birth incidence rate in Shenzhen, China, 2003-2012. *PROM-PTB*, preterm birth following premature rupture of membranes; *MI-PTB*, medically induced preterm birth; *S-PTB*, preterm birth due to spontaneous preterm labor; *APNCU*, the adequacy of prenatal care utilization index

The contributions of risk factor changes to differences in PTB rates are shown in Fig. 2. Larger proportions of younger and older women and higher educational attainment were associated with rising PTB rates, but lower proportions of inadequate prenatal care visits, and mothers with preeclampsia or eclampsia contributed to declining PTB rates, as did more multipara. The magnitudes of projected rate increases differed across PTB subtypes as shown by Additional file 1 Table S3. However, chronic conditions and gestational hypertension had only modest effects on increases of PTB rates. The increasing overall PTB was unexplained by the combined effect of changes in sociodemographic and pregnancy characteristics (projected − 0.11% rate change). In more detail, 12.9% (0.08% /0.62%) of changes for MI-PTB were explained by the risk factors considered, 25.0% (− 0. 10% /− 0. 40%) for S-PTB and 12.44% (0.02% /0.16%) for PROM-PTB, respectively.

## Discussion

This study highlighted the time trends of preterm birth incidence rate by subtype, and investigated the reasons behinds the changing rates in China. Our results demonstrated that PTB rates increased from 5.59% to 6.06% in Shenzhen, China, over the period 2003-2012, with high heterogeneity across three PTB subtypes. This increase predominantly took place in late preterm and MI-PTB, and the corresponding annual percent change (*APC*) were 1.34% and 4.19%, respectively. In stratified analyses according to maternal demographic characteristics, PTB rates rapidly increased in mothers with advanced age and high educational attainment, while these decreased in younger and mothers with low education. In addition, we found that higher educational attainment, adjusted for maternal age, increased PTB rates (projected increase rate = 0.20%), especially in MI-PTB.

### Increasing preterm birth rates and drivers behind the trends

The rising PTB incidence in Shenzhen was consistent with findings from Hubei, China, and also coincided with general trends worldwide [2, 9]. However, the Shenzhen increase (from 5.60% in 2003 to 6.06% in 2012) was slower than reported for Hubei (from 5.67% in 2001 to 10.5% in 2012) [9], but faster than for countries that successfully reduced PTB increase rates from 2001 to 2010, including Canada (from 7.4% in 2000 to 7.8% in 2010), New Zealand (from 7.4% in 2000 to 7.6% in 2010), and Lithuania (from 5.3% in 2000 to 5.4% in 2012) [7]. Different changes in PTB rates between geographic regions appear to be associated in part with prenatal care access [3, 8]. For example, in high-income regions, pregnant women were more likely to obtain sufficient prenatal care, which may decrease the risk of obstetric complications, including PTBs [3, 8]. Our findings, suggesting a decrease in PTB in association with adequate prenatal care, further corroborate the importance of prenatal and maternal care resources in determining PTB rates, in particular moderate and late PTB.

In terms of reasons for increasing PTB rates over time in Shenzhen, we found that elevated MI-PTB and late preterm played important roles in driving PTB rates. However, the incidences of maternal conditions were stable or declined during the study interval. This apparent contradiction may be due part to changing standards of clinical practice in China, that encourage obstetric intervention (e.g. caesarean deliveries), as reported by Chang et al. [7]. The multivariable analysis showed that changes in age and education among obstetric populations made important contributions to the increasing PTB rates. These findings suggest that cause of the rising PTB rates may be multifactorial, resulting from a higher number of high-risk pregnancies, coupled to more extensive implementation of reproductive interventions among older and more highly educated women, such as use of assisted reproductive technologies (ART) [20]. Unfortunately, data describing the use of ART services was not available for this analysis.

Except for the drivers for PTB rates, we found improving prenatal care was an important contributor to decreased PTB, especially in S-PTB. The strong contribution of prenatal care visits to PTB rate declines indicated that many women would have benefited from improved coverage of recommended basic antenatal care services [3]. In general, more opportunities for prenatal care exist with longer gestational age, potentially introducing reverse causation. To address this bias, we computed the APNCU index, which was standardized by gestational age to reflect the access to prenatal care. In China, a pregnant woman at least five prenatal care visits were recommended during pregnancy [21], while the international standard was 7-11 visits [16]. As a result, although proportions of inadequate prenatal care in China declined over time, 71.07% of mothers in our study received insufficient prenatal care. Hence, strengthening access to and delivery of prenatal care remains a critical strategy to help prevent PTB in China.

### Future research directions

Still, as for PTB trends in China, several important research questions remain to be answered in the future. First, PTB incidence rates increased in both Shenzhen and Hubei, but whether there were differences in trends between these single centers and China as a whole remains unclear. Second, although we found that changes in maternal age and education drove PTB rates, the pathways for these effects are unclear. Third, the cause of temporal trends in PTB rates is likely to be multifactorial, a result of changing risk factor incidences, clinical practices, and public policies, while few studies have assessed the contribution of these potential drivers.

### Strengths and limitations

Several previous studies, including a global estimation and two local investigations in China, have characterized recent trends in their rates of preterm birth overall [2, 9, 10]. Few studies, however, have documented the population-based temporal trends in PTB subtypes, and none has identified the determinants behind these trends. Rising PTB rates have been documented in Hubei, China, and the results of our study confirmed the increasing trends in a developed area of China. To our knowledge, this is the first study to characterize the time trends of PTB subtypes in mainland China, and to explore the reasons behind these trends.

The results of our study were limited by use of routinely collected registry data. This may have resulted in misclassified outcomes for some women. However, the high validity and reliability of the Shenzhen Birth Registry has been previously described [13], and an obstetrician reviewed each of the case records for accuracy, so we anticipate the impact was small. Next, we captured births only among women 13-50 years of age, and so may have missed PTB cases among higher risk ages. Yet, we defined the inclusion criterion according to the mean ages at menarche (12.76 years) and natural menopausal (50.76 years) in the Chinese population [22, 23], and so the impact is likely to have been modest. Third, in the analysis of determinants for PTBs, we also did not consider the association between cigarette smoking rates and PTB trends [24], as these data were unavailable in the birth registry. However, smoking prevalence has been stable in women according to the China National Health Services Survey [21]. A recent study showed that smoking prevalence was only 0.7% among women in Shenzhen [25], and so was unlikely to meaningfully bias our results.

## Conclusions

In summary, the Shenzhen findings confirm the previous reports of an increasing trend for PTB rates in mainland China. Moreover, this study also suggested that older and more highly educated pregnant woman, should be key target population groups for clinical PTB interventions and public health PTB prevention strategies in developed areas of China. However, considering the wide variation of PTB rates among geographic areas, we suggest caution in generalizing the Shenzhen findings.

Preterm birth rate increased in Shenzhen between 2003 and 2012, yet with varied trends among three PTB subtypes. Maternal age, education, parity and prenatal care visits played important roles in determining secular trends for PTB rates. These findings represent potential targets for interventions or policies designed to reduce PTB. More knowledge on how these factors are associated with PTB in China is needed for shaping future prevention strategies. Our findings also highlight the importance of adequate prenatal care for reducing PTB in China.

## Additional file

Additional file 1: **Figure S1**. Flowchart of final study population. **Table S1**. Temporal Trends in Preterm Birth Incidence Rates and Proportion of Preterm Birth Subtypes in Shenzhen, China, 2003-2012. **Table S2**. Sensitivity Analysis for Temporal Trends for Overall Preterm Birth Incidence Rates in Shenzhen, China, during 2003-2012. **Table S3**. Analysis of Factors Contributing to Changing Preterm Birth Incidence Rates in Shenzhen, 2003-2012. (DOCX 51 kb)

## Abbreviations

*AF*_*p*_: Population-attributable risk fraction
*AOR*s: Adjusted odds ratios
APC: Annual percent change
APNCU: The adequacy of prenatal care utilization
ART: Assisted reproductive technology
ICSI: Institute for Clinical Systems Improvement
LMP: Date of mother’s last menstrual period
MI-PTB: Medically induced preterm birth
Overall-PTB: All preterm births
PROM: following premature rupture of membranes
PTB: Preterm birth
*RR*: Rate ratios
S-PTB: Preterm birth due to spontaneous preterm labor
