# Supplementary material for: Temporal trends of preterm birth in Shenzhen, China: a retrospective study
Source: Reprod Health. 2018 Mar 13;15:47. doi: 10.1186/s12978-018-0477-8 (PMC5851155; doi:10.1186/s12978-018-0477-8)
Supplement: Supplementary file 1 — Figure S1. Flowchart of final study population. Table S1. Temporal Trends in Preterm Birth Incidence Rates and Proportion of Preterm Birth Subtypes in Shenzhen, China, 2003-2012. Table S2. Sensitivity Analysis for Temporal Trends for Overall Preterm Birth Incidence Rates in Shenzhen, China, during 2003-2012. Table S3. Analysis of Factors Contributing to Changing Preterm Birth Incidence Rates in Shenzhen, 2003-2012. (DOCX 51 kb) [file 12978_2018_477_MOESM1_ESM.docx]

| **Temporal Trends in Preterm Birth in Shenzhen, China: A Retrospective Study** |
| --- |
| **Supplementary Table of Contents** |
| **Figure A1. Flowchart of final study population……………………………………………………1** |
| Table A1. Temporal Trends in Preterm Birth Incidence Rates and Proportion of Preterm Birth Subtypes in Shenzhen, China, 2003-2012 **……**2 |
| Table A2. Sensitivity Analysis for Temporal Trends for Overall Preterm Birth Incidence Rates in Shenzhen, China, during 2003-2012 **………**3 |
| Table A3. Analysis of Factors Contributing to Changing Preterm Birth Incidence Rates in Shenzhen, 2003-2012 **……………………………**4 |

Births among women in the Shenzhen Birth Registration System

(From 1^st^ January, 2003 to 31^st^ December, 2012)

(n= 1,420,189)

Births included in the study population

(n= 1, 385, 882 )

Excluded (n= 34, 307):

- Still births (n=2135)
- Births to miss gestational age (n=261)
- Births to miss maternal age

(n=202)

- Births to mothers aged < 13 yr or > 50 yr

(n=26608)

- Births at < 22 wk or 46 wk gestation

(n=5101)

**Figure A1. Flowchart of final study population**

| **Table A1. Temporal Trends in Preterm Birth Incidence Rates and Proportion of Preterm Birth Subtypes in Shenzhen, China, 2003-2012** | | | | | | | | | | | |  |
| --- | --- | --- | --- | --- | --- | --- | --- | --- | --- | --- | --- | --- |
|  | **Year** | | | | | | | | | | **APC**^a^ **(%) (95% CI)** | |
|  | **2003** | **2004** | **2005** | **2006** | **2007** | **2008** | **2009** | **2010** | **2011** | **2012** |  |  |
| **No. of live births** | 56505 | 79108 | 90472 | 110137 | 142576 | 160466 | 158729 | 174232 | 187218 | 226379 | - | |
| **No. of preterm births** | 3157 | 4336 | 4863 | 5868 | 7540 | 8818 | 8724 | 10055 | 11179 | 13712 | - | |
| **Preterm birth rates (%)** | 5.59 | 5.48 | 5.38 | 5.33 | 5.29 | 5.49 | 5.50 | 5.77 | 5.97 | 6.06 | 0.94 ( 0.30, 1.58 ) | |
| **Subtypes-specific preterm birth proportions ^b^ (%)** |  |  |  |  |  |  |  |  |  |  |  | |
| PROM-PTB | 5.61 | 2.74 | 11.23 | 9.79 | 8.47 | 7.69 | 8.92 | 7.79 | 13.70 | 12.04 | 8.60 ( 1.45, 16.38 ) | |
| S-PTB | 64.71 | 62.57 | 57.60 | 56.31 | 55.41 | 54.85 | 49.34 | 49.16 | 43.50 | 45.56 | - 4.07 (-4.73, -3.40) | |
| MI-PTB | 29.68 | 34.69 | 31.17 | 33.91 | 36.11 | 37.45 | 41.75 | 43.05 | 42.80 | 42.40 | 4.19 ( 3.07, 5.32 ) | |
| **Subtypes-specific preterm birth rates (%)** |  |  |  |  |  |  |  |  |  |  |  | |
| PROM-PTB | 0.31 | 0.15 | 0.60 | 0.52 | 0.45 | 0.42 | 0.49 | 0.45 | 0.82 | 0.72 | 3.13 ( 1.01, 5.31 ) | |
| S-PTB | 3.62 | 3.43 | 3.10 | 3.00 | 2.93 | 3.01 | 2.71 | 2.84 | 2.59 | 2.74 | - 2.34 (-3.06, -1.62) | |
| MI-PTB | 1.66 | 1.90 | 1.68 | 1.81 | 1.91 | 2.06 | 2.29 | 2.48 | 2.55 | 2.55 | 3.60 ( 2.73, 4.48 ) | |
| **Preterm birth rates by gestational age (%)** |  |  |  |  |  |  |  |  |  |  |  | |
| < 32 weeks | 0.64 | 0.62 | 0.63 | 0.55 | 0.53 | 0.55 | 0.53 | 0.58 | 0.63 | 0.59 | -0.26 (-0.85, 3.48) | |
| 32-34 weeks | 0.75 | 0.78 | 0.72 | 0.71 | 0.73 | 0.74 | 0.68 | 0.68 | 0.74 | 0.73 | -0.26 (-0.63, 1.06) | |
| 35-36 weeks | 4.19 | 4.08 | 4.03 | 4.07 | 4.03 | 4.20 | 4.28 | 4.50 | 4.60 | 4.74 | 1.34 (0.75, 1.93) | |
| **Maternal age-specific preterm birth rates (%)** |  |  |  |  |  |  |  |  |  |  |  | |
| ≤20 y | 8.89 | 8.01 | 7.75 | 6.90 | 7.49 | 6.50 | 6.23 | 6.90 | 6.65 | 7.19 | -2.24 (-3.69, -0.77 ) | |
| 21-35 y | 5.41 | 5.31 | 5.17 | 5.13 | 5.03 | 5.25 | 5.23 | 5.47 | 5.72 | 5.78 | 0.77 ( 0.07, 1.48 ) | |
| ≥36 y | 6.76 | 7.43 | 7.20 | 7.18 | 7.26 | 8.08 | 8.46 | 9.06 | 9.10 | 9.34 | 3.33 ( 2.54, 4.12 ) | |
| **Maternal education-specific preterm birth rates (%)** |  |  |  |  |  |  |  |  |  |  |  | |
| Less than high school | 6.25 | 6.05 | 5.84 | 5.69 | 5.74 | 5.64 | 5.41 | 5.55 | 5.60 | 5.85 | - 0.80 (-1.41, -0.20) | |
| High school and college | 5.24 | 5.27 | 5.03 | 5.13 | 4.98 | 5.60 | 5.91 | 6.23 | 6.26 | 6.38 | 2.39 ( 1.44, 3.35 ) | |
| Bachelor | 3.80 | 4.36 | 4.66 | 4.66 | 4.63 | 4.85 | 5.11 | 5.45 | 5.94 | 5.78 | 3.60 ( 2.85, 4.36 ) | |
| Postgraduate | 2.56 | 4.91 | 4.05 | 3.94 | 4.71 | 5.87 | 5.20 | 5.25 | 7.08 | 5.41 | 5.87 ( 2.56, 9.29 ) | |

^a^ APC, the annual percent change; CI, confidence interval;

^b^ PROM-PTB, preterm birth following premature rupture of membranes; MI-PTB, medically induced preterm birth; S-PTB, preterm birth due to spontaneous preterm labor.

| **Table A2. Sensitivity Analysis for Temporal Trends for Overall Preterm Birth Incidence Rates**  **in Shenzhen, China, during 2003-2012** | | |
| --- | --- | --- |
| **Years ^a^** | **Risk Ratios (95% CI)** | ***P* value** |
| Reference: 2003 |  |  |
| 2004 | 0.98 (0.72, 7.98) | 0.90 |
| 2005 | 1.19 (0.88, 12.04) | 0.26 |
| 2006 | 1.16 (0.86, 11.35) | 0.34 |
| 2007 | 1.28 (0.94, 14.36) | 0.11 |
| 2008 | 1.53 (1.13, 23.45) | 0.01 |
| 2009 | 1.51 (1.11, 22.54) | 0.01 |
| 2010 | 1.73 (1.27, 34.7) | 0.00 |
| 2011 | 1.29 (0.95, 14.65) | 0.10 |
| 2012 | 1.45 (1.07, 20.04) | 0.02 |

| **Table A3. Analysis of Factors Contributing to Changing Preterm Birth Incidence Rates in Shenzhen, 2003-2012 ^a^** | | | | | | | | | | | |
| --- | --- | --- | --- | --- | --- | --- | --- | --- | --- | --- | --- |
|  | **Overall PTB** | |  | **PROM-PTB** | |  | **S-PTB** | |  | **MI-PTB** | |
|  | ***AOR*** | ***Projected increase* ^b^ *(%)*** |  | ***AOR*** | ***Projected increase* ^b^ *(%)*** |  | ***AOR*** | ***Projected increase* ^b^ *(%)*** |  | ***AOR*** | ***Projected increase* ^b^ *(%)*** |
| **Maternal age** (*years*) |  |  |  |  |  |  |  |  |  |  |  |
| 21-35 | Reference | 0.04 |  | Reference | 0.00 |  | Reference | 0.03 |  | Reference | 0.01 |
| ≤20 | 1.07 |  |  | 0.62 |  |  | 1.50 |  |  | 0.53 |  |
| ≥36 | 1.62 |  |  | 2.06 |  |  | 0.99 |  |  | 2.28 |  |
| **Maternal education** |  |  |  |  |  |  |  |  |  |  |  |
| Bachelor | Reference | 0.20 |  | Reference | 0.06 |  | Reference | 0.00 |  | Reference | 0.17 |
| Less than high school | 0.70 |  |  | 0.43 |  |  | 0.97 |  |  | 0.55 |  |
| High school and college | 0.85 |  |  | 0.72 |  |  | 1.00 |  |  | 0.81 |  |
| Postgraduate | 1.00 |  |  | 0.82 |  |  | 0.86 |  |  | - |  |
| **Parity** (*times*) |  |  |  |  |  |  |  |  |  |  |  |
| 0 | Reference | -0.08 |  | Reference | -0.02 |  | Reference | -0.04 |  | 1.00 | -0.02 |
| ≥1 | 0.80 |  |  | 0.57 |  |  | 0.81 |  |  | 0.86 |  |
| Missing data | - |  |  | - |  |  | - |  |  |  |  |
| **APNCU index** |  |  |  |  |  |  |  |  |  |  |  |
| Appropriate | Reference | -0.15 |  | Reference | -0.01 |  | Reference | -0.10 |  | Reference | 0.01 |
| Inadequate | 1.81 |  |  | 1.50 |  |  | 3.19 |  |  | 1.14 |  |
| Intermediate | 1.34 |  |  | 1.27 |  |  | 1.88 |  |  | 1.11 |  |
| Appropriate plus | 0.44 |  |  | 0.35 |  |  | 0.26 |  |  | 0.53 |  |
| Missing data | - |  |  | - |  |  | - |  |  | - |  |
| **Maternal chronic conditions** |  |  |  |  |  |  |  |  |  |  |  |
| No | Reference | 0.01 |  | Reference | 0.00 |  | Reference | 0.00 |  | Reference | 0.00 |
| Yes | 3.14 |  |  | 6.40 |  |  | 1.25 |  |  | 3.76 |  |
| **Gestational hypertension** |  |  |  |  |  |  |  |  |  |  |  |
| No | Reference | 0.01 |  | Reference | 0.00 |  | Reference | 0.00 |  | Reference | 0.01 |
| Yes | 1.62 |  |  | 2.58 |  |  | 0.70 |  |  | 2.22 |  |
| **Preeclampsia or eclampsia** |  |  |  |  |  |  |  |  |  |  |  |
| No | Reference | -0.05 |  | Reference | 0.00 |  | Reference | 0.00 |  | Reference | -0.05 |
| Yes | 5.40 |  |  | - |  |  | 0.65 |  |  | 12.37 |  |
| **Infant sex** |  |  |  |  |  |  |  |  |  |  |  |
| Female | Reference | -0.01 |  | Reference | 0.00 |  | Reference | 0.00 |  | Reference | 0.00 |
| Male | 1.15 |  |  | 1.17 |  |  | 1.21 |  |  | 1.07 |  |
| Hermaphrodite | 3.37 |  |  | - |  |  | 5.94 |  |  | 1.00 |  |
| ***Projected increase (%)*** | - | -0.11 |  | - | 0.02 |  | - | -0.10 |  | - | 0.08 |

^a^ PROM-PTB, preterm birth following premature rupture of membranes; MI-PTB, medically induced preterm birth; S-PTB, preterm birth due to spontaneous preterm labor.

^b^ Projected increase: Difference of population attributed risk from 2008-2012 baseline preterm birth rate with distribution of specific risk factors during 2003-2007 and 2008-2012.
